# Involvement of extracellular vesicles in the progression, diagnosis, treatment, and prevention of whole-body ionizing radiation-induced immune dysfunction

**DOI:** 10.3389/fimmu.2023.1188830

**Published:** 2023-06-15

**Authors:** Roland F. Seim, Laura E. Herring, Angie L. Mordant, Micah L. Willis, Shannon M. Wallet, Leon G. Coleman, Robert Maile

**Affiliations:** ^1^ Curriculum in Toxicology & Environmental Medicine, University of North Carolina at Chapel Hill, Chapel Hill, NC, United States; ^2^ Department of Pharmacology, University of North Carolina at Chapel Hill School of Medicine, Chapel Hill, NC, United States; ^3^ Department of Oral Biology, University of Florida, Gainesville, FL, United States; ^4^ Department of Surgery, University of Florida, Gainesville, FL, United States

**Keywords:** ionizing radiation, extracellular vesicles, mesenchymal stem cells, burn injury, radiation syndrome, immune dysfunction

## Abstract

Acute radiation syndrome (ARS) develops after exposure to high doses of ionizing radiation and features immune suppression and organ failure. Currently, there are no diagnostics to identify the occurrence or severity of exposure and there are limited treatments and preventative strategies to mitigate ARS. Extracellular vesicles (EVs) are mediators of intercellular communication that contribute to immune dysfunction across many diseases. We investigated if EV cargo can identify whole body irradiation (WBIR) exposure and if EVs promote ARS immune dysfunction. We hypothesized that beneficial EVs derived from mesenchymal stem cells (MSC-EVs) would blunt ARS immune dysfunction and might serve as prophylactic radioprotectants. Mice received WBIR (2 or 9 Gy) with assessment of EVs at 3 and 7 days after exposure. LC-MS/MS proteomic analysis of WBIR-EVs found dose-related changes as well as candidate proteins that were increased with both doses and timepoints (34 total) such as Thromboxane-A Synthase and lymphocyte cytosolic protein 2. Suprabasin and Sarcalumenin were increased only after 9 Gy suggesting these proteins may indicate high dose/lethal exposure. Analysis of EV miRNAs identified miR-376 and miR-136, which were increased up to 200- and 60-fold respectively by both doses of WBIR and select miRNAs such as miR-1839 and miR-664 were increased only with 9 Gy. WBIR-EVs (9 Gy) were biologically active and blunted immune responses to LPS in RAW264.7 macrophages, inhibiting canonical signaling pathways associated with wound healing and phagosome formation. When given 3 days after exposure, MSC-EVs slightly modified immune gene expression changes in the spleens of mice in response to WBIR and in a combined radiation plus burn injury exposure (RCI). MSC-EVs normalized the expression of certain key immune genes such as *NFκBia* and *Cxcr4* (WBIR), *Map4k1*, *Ccr9* and *Cxcl12* (RCI) and lowered plasma TNFα cytokine levels after RCI. When given prophylactically (24 and 3 hours before exposure), MSC-EVs prolonged survival to the 9 Gy lethal exposure. Thus, EVs are important participants in ARS. EV cargo might be used to diagnose WBIR exposure, and MSC-EVs might serve as radioprotectants to blunt the impact of toxic radiation exposure.

## Introduction

The likelihood of a radiological incident occurring in the general population is growing due to the increased reliance on nuclear power, the risk of sophisticated terrorist attacks, and the current threats of nuclear warfare (1, 2). Such exposures can result in the development of Acute Radiation Syndrome (ARS) in affected individuals that are exposed to high doses of radiation over most or the entire body within a short period of time (3). Exposure to whole-body ionizing radiation (WBIR) impacts all organ systems, with rapid induction of a systemic inflammatory response, mediated by many mechanisms such as induction of the Acute Phase Response (APR) and peripheral and central (bone marrow) cell death; however, rapidly dividing cells are the most radiosensitive (4). Thus, the three main ARS syndromes exist: hematopoietic, gastrointestinal, and neurovascular (3). After exposure to 2 grays (Gy), bone marrow cells are depleted, resulting in death after about 30-60 days (3). Thus, peripheral immune function is greatly impaired (5, 6). Similar ARS syndromes occur in rodents, though higher doses are required than in humans for mortality (~9 Gy) (7, 8). Since symptoms can take days or weeks to develop after exposure, it can be difficult to determine if individuals have been exposed and their level of exposure. There are also delayed effects of acute radiation exposure (DEARE) resulting in multiple chronic conditions affecting multiple organ systems and bacterial susceptibility (9), and it is thought that reducing the amplitude of ARS can also reduce DEARE. There is also a pressing need for field-deployable approaches to detect and determine radiation dose after a large-scale radiological event using an assay that is as minimally invasive as possible. Therefore, given the increasing risks of WBIR exposure and its profound biological consequences, research gaps include a lack of biomarkers to identify radiation exposure, and a corresponding paucity of radiation medical countermeasures (MCM) that can act as mitigators of acute (and chronic) radiation-induced immune dysfunction. In addition, as we have described, radiation and polytrauma models share a common theme, with increased tissue damage and resultant signals driving the central immune dysfunction leading to increased infection susceptibility and aberrant wound healing (10–12). Polytrauma patients have a greater amplitude of dysfunctional responses. Indeed, radiation combined with burn injury (RCI) causes a more severe ARS and occurs when patients are exposed to high doses of radiation in addition to burn injury. Historically, 65-70% of the survivors of a nuclear incident will have significant burn injuries in addition to exposure to high doses of radiation (13). RCI results in a higher level of lethality and the exacerbation of physiological complications associated with either burn or radiation alone (14, 15). Due to the severity of RCI, there is an extremely intense inflammatory response early after injury that is one of the major contributors to the high mortality rate observed in people (16). We have previously described a pre-clinical mouse model of RCI that exhibits these phenotypes and utilized this model to understand the cellular and molecular elements that exist to control immune recovery after polytrauma such as RCI (10, 12).

A key aspect of radiation injury is the “Bystander Effect”, wherein irradiated cells transmit signals that can cause damage to non-irradiated cells (17, 18). Nagasawa found that irradiating 1% of cells would cause DNA damage in more than 30% of other nearby cells. Further, serum isolated from Chernobyl survivors was found to cause chromosomal damage in cultured cells (9), with effects that can last for up to 30 years after the initial exposure (19). The bystander effect can result in genomic instability, cellular stress responses, oxidative damage, apoptosis, and immune activation (18, 20). The secretion of clastogenic factors, cytokines, damage-associated molecular patterns, miRNAs, and lipid rafts may contribute (20). Extracellular vesicles (EVs) are multimodal signaling mediators that carry these factors, and are implicated in immune dysfunction in various settings (18, 21). We hypothesized that EVs contribute to post-WBIR immune dysfunction.

EVs are phospholipid enclosed structures secreted by almost all cell types. EVs transport a diverse array of cargo that includes miRNA, DNA, histones, long non-coding RNAs, proteins, lipids, and both damage associated molecular patterns (DAMPs) and pathogen associated molecular patterns (PAMPs). Due to their ability to harbor a diverse array of cargo, EVs can induce powerful and complex biological effects on downstream or recipient cells. They have also been identified as critical mediators across a wide range of pathologies, including traumatic injuries, central nervous system disorders, cancer, autoimmune diseases, and cardiovascular disease (21–25). In the context of radiation, recent attention has been placed on EVs as one of the contributors of the Bystander Effect (18, 26). EVs released from irradiated cells caused chromosomal damage in naïve cells (27), and transfer of EVs isolated from mice exposed to WBIR to naïve mice induced immune signaling changes that were comparable to the effects of direct radiation (18). However, the influence of WBIR on plasma EV cargo is unknown, as well as the ability of EVs to be used as biomarkers of WBIR. We hypothesized that EV protein and miRNA cargo would be impacted by WBIR and could be used to inform the level of radiation in exposed individuals.

Given the likely detrimental role of EVs in ARS, it is reasonable to posit that administration of protective EVs might improve symptoms. Mesenchymal stem cell-derived EVs (MSC-EVs) carry trophic and immunomodulatory signals that have shown therapeutic benefit across a range of diseases (21). Administration of MSC-EVs after WBIR have been found to slow the progression of ARS (28–30). However, the impact of MSC-EV treatment on immune dysfunction is unknown. Further, it is unknown whether MSC-EVs could be given prophylactically as a radioprotectant to prevent ARS in those at increased risk for exposure. This would be a valuable tool that could be administered to soldiers, emergency personnel, and nuclear power-plant workers during radiological attacks or incidents (31). In addition, we further utilized our murine model of RCI (10, 12) to evaluate if treating mice with mesenchymal-stem cell derived extracellular vesicles (MSC-EVs) could improve immune outcomes due to their inherent regenerative and anti-inflammatory properties (32). We hypothesized that MSC-EVs would improve immune dysfunction caused by WBIR and RCI, and that MSC-EV administration prior to WBIR would reduce ARS.

## Materials and methods

### Mouse irradiation injury model

The protocols described here were performed in accordance with the Guide for the Care and Use of Laboratory Animals of the National Institute of Health. This protocol was approved by the University of North Carolina Institutional Animal Care and Use Committee with ethically approved experimental design. All animals were housed in an American Association for Accreditation of Laboratory Animal Care accredited facility with full time veterinary staff. All animals were monitored closely throughout the duration of the experiments.

Female C57BL/6J mice between 6-8 weeks of age with a weight of 15-20g were used for all experiments. Mice were placed in a mouse pie cage before receiving either 2 Gy or 9 Gy of radiation (dose rate of about 0.8 Gy/min) from a Cs^137^ based irradiator developed by Best Theratronics Ltd. (Kanata, Ontario). After the irradiation procedure, mice were returned to their cages and monitored closely following the procedure. They were provided with food and water ad lib throughout the procedure and if the mice showed any overt symptoms of radiation sickness (hunched, dehydrated, difficulty breathing, loss of > 20% body weight, inactivity, or lesions), then they were euthanized immediately with inhaled isoflurane (drop method), followed by cervical dislocation.

### Mouse model of combined irradiation and burn injury model

The model of murine burn injury combined with irradiation has been previously described (10, 12). Female C57BL/6J mice between 6-8 weeks of age with a weight of 15-20g were used for all experiments. Briefly, for the burn injury procedure, mice were anesthetized with tribromoethanol (avertin; 475 mg/kg) and the dorsum and flank of the mouse was shaved, and morphine sulfate (3mg/kg) was injected subcutaneously into the dorsum of the mouse. Following injection and anesthesia, a copper rod, heated to 100°C by a boiling water bath, was applied to the dorsum and flank of the mouse for ten seconds. This was repeated four times with a 65g copper rod (1.9 cm in diameter) to achieve a full-thickness contact burn of 20% total body surface area (TBSA). After the burn procedure, mice were resuscitated with an intraperitoneal injection of Lactated Ringer’s solution (0.1 mL/g of body weight). Within one hour of the burn procedure, the mice were exposed to 9 Gy of WBI from a Cs^137^ (dose rate of about 0.8 Gy/min) based irradiator developed by Best Theratronics Ltd (Kanata, Ontario). Sham groups went through an identical procedure but were not burned or irradiated. The mice were returned to individual cages and given food and morphinated water *ad lib*. The mice were monitored twice daily throughout the experiments and if mice developed overt symptoms of injury that could not be easily treated (dehydration, hunched posture, difficulty breathing, >20% body weight loss, or inactivity), then the mice were immediately euthanized utilizing inhaled isoflurane (drop method) followed by cervical dislocation. Following exposure to WBIR or radiation combined with burn injury (RCI), the plasma and spleen was harvested from these mice and stored at -80°C before further processing.

### Extracellular vesicle isolation, quantification, and sizing

EVs were isolated from the plasma of mice 3 days and 7 days following exposure to either 2 Gy or 9 Gy of WBIR *via* differential centrifugation as previously described (33–35). Mice were euthanized by inhaled isoflurane and blood was collected *via* cardiac puncture and collected in tubes containing 40% trisodium citrate. The plasma was centrifuged for 2000xg for 20 min to remove cells. Following this, the supernatant was collected and spun at 10,000xg for 30 min to remove cellular debris. Finally, the supernatant from this spin was removed and spun at 21,000xg for 1 hr to pellet EVs. The EV containing-pellet was resuspended in 30 µl of phosphate buffered saline (PBS) that was filtered with a Whatman™ 0.02 µm syringe filter and frozen at -80°C before analysis. To assess the quality and concentration of our isolations, Nanoparticle Tracking Analysis (NTA) was performed on the final EV products using the ZetaView QUATT instrument (Particle Metrix, Mebane, NC) and ZetaView (version 8.05) software. The EV pellets were diluted 1:1000 in 0.02 µm syringe filtered PBS. The mean concentrations (EV/ml) and size were determined by taking 10 videos with a 488 nm laser, pump speed 30, camera shutter of 100. Each measurement from the videos was screened for quality control and all videos that failed were excluded.

### Unbiased proteomic assessment of EVs isolated from mice following WBIR

EVs were isolated from mice exposed to either 2 Gy and 9 Gy of WBIR 3 days and 7 days following exposure as well as sham (uninjured) mice and prepared for unbiased proteomic assessment using LC-MS/MS (36). Following the last spin of EVs, the EV-containing pellet was resuspended in 20 mM Tris buffer (pH 7.5). Next, 8 M Urea was added to the protein samples (about 10-20 µg per sample), then reduced with 5mM dithiothreitol (DTT) for 30 min. After reduction, the samples were alkylated with 15 mM iodoacetamide for 45 minutes. The samples were then diluted with 1 M urea before digestion with mass-spec grade Trypsin (Promega, Madison, WI) at 37°C overnight. Following the overnight incubation, the peptide samples were acidified with 1% trifluoroacetic acid (TFA) before desalting with Pierce™ C18 spin columns (ThermoFisher Scientific, Waltham, MA). Peptide quantification was then performed utilizing a Pierce™ bicinchoninic acid assay (BCA) fluorometric peptide quantitation assay. The samples were dried *via* vacuum centrifugation and resuspended in 0.1% formic acid. Samples were normalized to 0.1 µg/µl. Pooled samples were used to assess technical reproducibility and were prepared by combining a small portion of each sample. 0.5 µg of sample was analyzed by LC-MS/MS using a ThermoFisher Easy nLC 1200 coupled to a QExactive HF (ThermoFisher Scientific, Waltham, MA) with an Easy Spray PepMap C18 column (75 μm id × 25 cm, 2 μm particle size) (ThermoFisher Scientific, Waltham, MA). The samples were separated over a 90-minute period where the gradient of separation consisted of 5-32% mobile phase B kept at a flow rate of 250nL/min and a mobile phase A consisting of 0.1% formic acid in acetonitrile. The QExactive HF identified the 15 most intense precursors and selected them for subsequent Higher-energy C-trap dissociation (HCD). For the precursor scan, the resolution was set to 60,000 with a target value of 3 x 10^6^ ions, 100 ms inject time; for MS2 scan, the resolution was set to 15,000 with a target value of 1 x 10^5^ ions, 75 ms inject time. The collision energy set to 27% for the HCD, with an isolation window of 1.6 *m/z.* Peptide match was set to preferred and the precursors with an unknown charge or a charge state of 1 and ≥7 were excluded. The proteins were identified and quantified with Proteome Discoverer 2.5 utilizing a Uniport Mouse database (~ 17,000 sequences). The peptide false discovery rate (FDR) was set to 1% and only proteins with >1 peptide were used for downstream analyses. Proteins were media-normalized within Proteome Discoverer. The level of lcp2 protein was also measured by ELISA (MyBioSource) according to the manufacturer’s instructions as we have done previously (36).

### Transmission electron microscopy of EVs

To visualize EVs, isolated EVs were prepared for negative-stain transmission electron microscopy. A glow-discharged formvar/carbon-coated 400 mesh copper grids (Ted Pella, Inc., Redding, CA) was floated on a droplet of the sample suspension for 12 minutes, transferred quickly to 2 drops of deionized water followed by a droplet of 2% aqueous uranyl acetate stain for 1 minute. The grid was blotted with filter paper and air-dried. Samples were observed using a JEOL JEM-1230 transmission electron microscope operating at 80kV (JEOL USA INC., Peabody, MA) and images were taken using a Gatan Orius SC1000 CCD camera with Gatan Microscopy Suite version 3.10.1002.0 software (Gatan, Inc., Pleasanton, CA).

### Cytokine and chemokine detection

Bio-Plex Immunoassays (Hercules, CA, USA) were utilized to analyze the cytokine/chemokine levels of TNF-α, IL-2, and MCP-1 according to the manufacturers protocols in the mouse plasma following RCI. The data was acquired on a Bio-plex 200 system with Bio-Plex Manager and Bio-Plex Pro Software and analyzed using a five-parameter logistic spline-curve fitting method. The data are presented as picograms/ml.

### RAW 264.7 cell culture and EV exposure

RAW 264.7 (ATCC, Manassas, VA, USA) mouse macrophage cells were grown in culture according to the manufacturer’s recommendations. The cells were cultured in Dulbecco’s Modified Eagle Medium (DMEM) containing 10% Fetal Bovine Serum (FBS) and 1% penicillin/streptomycin at 37°C and 5% C0_2_. For the EV exposure, a total of 1x10^6^ cells were plated in a 24-well plate and allowed to adhere overnight. The following day, 1x10^7^ EVs were added to the cells in the presence of 1 µg/ml lipopolysaccharide (LPS) from *Escherichia coli* 0111: B4 for 24 hr. Cellular mRNA was harvested for analysis.

### C57BL/6J mouse mesenchymal stem cells culture, EV isolation, and *in vivo* transfers

C57BL/6J mouse bone marrow mesenchymal stem cells (MSCs) (Cyagen, Santa Clara, CA, USA) were grown in OriCell™ MSC Growth Medium (Cyagen, Santa Clara, CA, USA) containing 10% FBS and 1% penicillin/streptomycin at 37°C and 5% CO_2_ according to the manufacturer’s recommendations. MSCs were allowed to grow for three days before the media was removed and centrifuged at 2000xg for 20 min to remove cells. The supernatant was collected, and the media was spun at 10,000xg for 30 min to remove debris. Lastly, the supernatant was centrifuged at 21,000xg for 1 hr to pellet EVs and the supernatant was removed. The pellet was resuspended in 30 µl of phosphate buffered saline (PBS) that was filtered with a 0.02 µm syringe filter and frozen at -80°C before analysis. To assess the quality and concentration of our isolations, Nanoparticle Tracking Analysis (NTA) was performed on the final EV products using the ZetaView QUATT instrument (Particle Metrix, Mebane, NC) and ZetaView (version 8.05). Samples were concentrated to 1x10^10^ EVs and intravenously injected into the mice prior to WBIR or after RCI and WBIR.

### RNA isolation and immune gene quantification

RNA was isolated using Qiagen’s (Hilden, Germany) RNeasy kit. RAW 264.7 cells were lysed with RLT lysis buffer and processed using the RNeasy kit. Cell suspensions were prepared from the spleens of mice. Red blood cells were lysed with ACK Lysis Buffer (0.15M NH_4_Cl_4_, 1mM KHCO_3_, and 0.1 Mm Na_2_ EDTA in water) before lysing with RLT lysis buffer and processed using the RNeasy kit. Total RNA was quantified using a nanodrop 2000 spectrophotometer (Waltham, MA). NanoString Technology and the nCounter Mouse Immunology Panel (Nanostring, Seattle, WA) was used to assess 561 mRNAs with relevance to immune function (37). All samples were run in triplicate. 100ng of mRNA was hybridized to report-capture probe pairs (CodeSets) at 65°C for 16hrs. After hybridization, the nCounter Prep Station was used to process the samples. During this stage, excess probe was removed, aligned with the probe/target complexes, and these complexes were immobilized in the nCounter cartridge. The nCounter cartridge was placed in a digital analyzer for image acquisition and data processing. The expression levels of each gene was analyzed by quantifying the number of times the color-coded barcode was detected for each gene. For data analysis, nSolver V4.0 was utilized to normalize the data and calculate fold changes, the resulting ratios, and differential expression. The resulting data was analyzed using Ingenuity Pathway Analysis (IPA) software to identify pathway-specific responses.

### miRNA analysis

To assess changes in the miRNA content of EVs following exposure to WBIR, nanoString technology and the nCounter mouse miRNA panel v2 that allows for the evaluation of 577 miRNAs was used (38). Following differential centrifugation, the EV pellets were disrupted using Qiazol Lysis Reagent (Qiagen, Hilden, Germany) and the miRNA easy kit was used for the isolation of mRNA and miRNA. Nanodrop ND1000 (NanoDrop Technologies, Waltham, MA) was used to assess the quantity and quality (A260/280 and A260/A230) of mRNA. A total of 100 ng of mRNA was used for the mouse nanoString nCounter miRNA microarray assay according to the manufacturer’s instructions. Briefly, the miRNAs were hybridized to probes at 65°C for 30 hrs. Afterwards, the hybridized probes were extended and quantified using the nCounter Prep Station and Digital Analyzer. The data was analyzed using the nSolver 4.0 software based on the manufacturer’s instructions for analyzing miRNA data.

### Phagocytosis assay

For the phagocytosis assay, 2.5x10^5^ RAW cells were plated in a 96-well flat bottom plate and were allowed to adhere for one hour. Utilizing the Vybrant™ (Thermo Fisher Scientific) phagocytosis assay kit, the cells were subsequently exposed to killed *E.coli* (K-12 strain), which were labelled with fluorescein in the presence of LPS from *Escherichia coli* 0111:B4 (10 μg/ml). Phagocytosis occurred for 2 hrs before aspirating the extracellular fluorescent *E. coli* and quenching the reaction in trypan blue. The intracellular fluorescence was quantified at an excitation of 480 nm and 520 nm emissions using a BioMek plate reader. In accordance with the manufacturer’s instructions, we subtracted the average fluorescence units of no-cell negative-control wells from all wells. We then defined phagocytosis response to the experimental effector (% Effect) as: % Effect = Net experimental phagocytosis × 100% x Net positive control phagocytosis.

### Statistical analysis

For the proteomics, the UNC Mass Spectrometry Core handled the date processing and statistical analyses. Briefly, the raw data files were processed using Proteome Discoverer 2.5 and searched against the Mouse Uniprot database (containing 16,940 sequences) (39). Trypsin was specified as the enzyme and only up to two missed cleavage sites were allowed. The carbamidomethylation of Cys was set as the fixed modification and oxidation of Met was used for variable modification. A 1% false discovery rate (FDR) was used to filter data and label-free quantification (LFQ) of unique peptides was used. At minimum, there had to be 2 unique peptides per protein and >50% non-zero values across all data sets were essential for all quantification. Further data analysis was conducted in Perseus (Gene Ontology Cellular Component was used for the annotation and imputation) and Argonaut was used for Log2 transformation and statistical tests (40). For all of the nanoString analyses, nSolver v4.0 was used to normalize the miRNA and mRNA fold changes to reveal ratios and differential expression data. Ingenuity Pathway Analysis was used to identify canonical pathways that were impacted and Z-scores greater than 2.0 and p-values <0.05 were considered to be significant (37). For the rest of the analyses, One-way ANOVAs with Dunnett’s *post-hoc* test were performed in GraphPad Prism version 9.0 for Windows. Data is displayed as mean ± standard error of the mean (*P<0.05, P**<0.01, and ***P<0.001).

## Results

### Whole body irradiation alters EV numbers in a dose dependent manner

**Figure 1:**
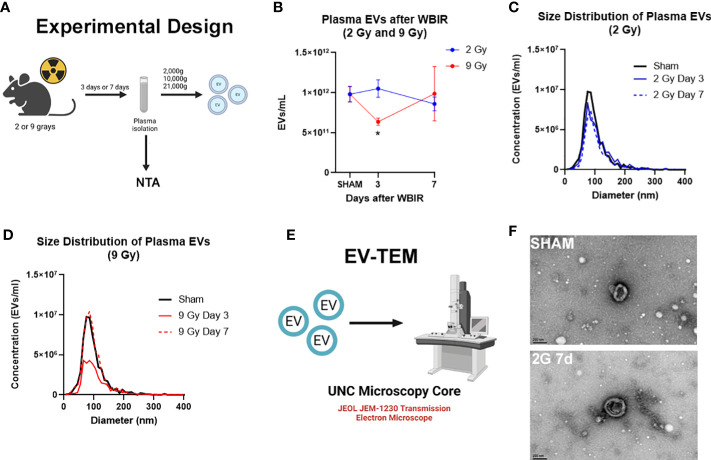
Total concentration of plasma extracellular vesicles are altered following WBIR. Assessing the plasma concentrations of extracellular vesicles (EVs) and confirmation that EVs were isolated following exposure to either 2 Gy or 9 Gy of whole-body ionizing radiation (WBIR). **(A)** Experimental design. C57BL/6 mice were exposed to either 2 Gy or 9 Gy of whole-body ionizing radiation from a Cesium-137 irradiator. EVs were isolated from the plasma of these mice 3 and 7 days after exposure (n=6 for each group). **(B-D)** Nanoparticle Tracking analysis (NTA) was used to measure the frequency and size distribution of EVs isolated following exposure to WBIR. **(B)** NTA found a reduction in plasma EVs at 3 days after WBIR. *p<0.05. One-way ANOVA with Dunnett’s *post-hoc* test. **(C)** Size Distribution of Plasma EVs at 3 and 7 days after 2 Gy exposure showed no shifts in the size of the EV pools. **(D)** Size Distribution of Plasma EVs at 3 and 7 days after 9 Gy exposure display a decrease in total EVs 3 Days after exposure to 9 Gy but there were not any shifts in the size distribution of these EVs **(E)** Approach for assessment of EVs by Transmission Electron Microscopy (TEM). **(F)** Representative TEM micrographs of plasma EVs isolated following sham or exposure to 2 Gy WBIR. Scale bar = 200nm. Created with BioRender.com.

**Figure 2:**
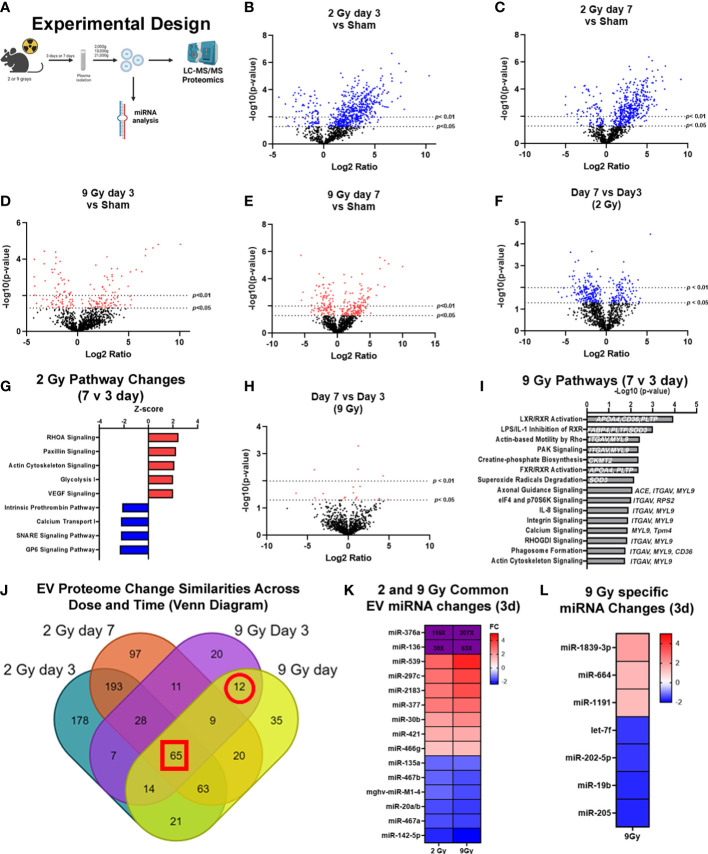
Exposure to WBIR induces alteration in the proteomic cargo of extracellular vesicles in a dose and time-dependent manner. **(A)** Experimental overview for the proteomic analysis of EVs isolated following WBIR. EVs were isolated from the plasma of mice 3 days and 7 days after exposure to 2 and 9 grays of WBIR. Protein content was measured by LC-MS/MS and all doses and treatment groups were compared to sham (uninjured) mice (n=3 for each experimental group). **(B)** Differential protein expression changes between EVs isolated 3 days after 2 Gy of WBIR compared to sham mice. **(C)** Differential protein expression changes between EVs isolated 7 days after 2 Gy of WBIR compared to sham mice. **(D)** Differential protein expression changes between EVs isolated 7 days after 9 Gy of WBIR compared to sham mice. **(E)** Differential protein expression changes between EVs isolated 7 days after 9 Gy of WBIR compared to sham mice. **(F)** Temporal changes in the proteomic cargo of these EVs following exposure to 2 Gy of WBIR. **(G)** Ingenuity Pathway Analysis (IPA) displaying the most predicted pathways to be activated or inhibited and associated Z-scores from day 7 to day 3 following exposure to 2 Gy of WBIR. **(H)** Temporal change in the protein cargo from day 7 to day 3 following exposure to 9 Gy of WBIR. **(I)** IPA analyses displaying the -Log10(p-values) of pathways that are predicted to be impacted from day 7 to day 3 following exposure to 9 Gy of WBIR. **(J)** Venn diagram displaying the overlap in significantly altered proteins between groups. **(K)** Heat map displaying significant fold changes in the miRNA cargo of EVs isolated 3 days after exposure to 2 and 9 Gy of WBIR. **(L)** Heat map displaying the significant miRNA alterations that were specific to EVs isolated 3 days after 9 Gy of WBIR. Created with BioRender.com.

Multiple studies have found changes in the number of circulating EVs after traumatic injuries (21, 41, 42). In order to determine the impact of radiation dose and time after injury on circulating EV numbers and size, mice received either 2 or 9 Gy of WBIR with sacrifice at 3 or 7 days after exposure (**Figure 1A**), which are moderate/survivable and lethal doses for mice respectively (8, 18). The total number and average diameter of the plasma EVs was measured by NTA. 2 Gy of WBIR had no impact on total EV number or size distribution up to 7 days after exposure compared to sham mice (**Figures 1B, C**). The total EV concentration was significantly reduced, however, 3 days after exposure to 9 Gy (**Figures 1B, D**). At 7 days after WBIR, there were no significant differences in total EV concentrations with either dose (**Figure 1B**). EVs were then isolated by sequential centrifugation which results in reliable isolation of ~0.05-500nm EVs (exosomes and microvesicles), with characteristic EV size and markers that we have measured by transmission electron microscopy (TEM), NTA, western blot, and flow cytometry (34, 36, 38, 43–45). Here, we confirmed the isolation of characteristically shaped EVs using TEM (**Figures 1E, F**). Next, we assessed EV protein and miRNA content using LC-MS/MS and nanoString analyses (**Figure 2A**). These data suggests that in our mouse model of WBIR, circulating EV concentrations are dependent on WBIR dose and time after exposure.

### Whole body irradiation alters protein and miRNA cargo in a dose and time-dependent manner

In order to determine the impact of radiation dose and time after injury on EV cargo, and therefore test the usefulness of EV as a source of “radiosensitive” biomarkers as outlined in **Figure 2A**, we assessed EV protein (**Figures 2B–J**) and miRNA (**Figures 2K, L**) content using LC-MS/MS and nanoString analyses, respectively. For the proteomic analysis, we found that across both doses and timepoints, exposure to WBIR significantly altered EV protein cargo compared to sham mice (**Figures 2B–F**). Two Gy of WBIR caused robust changes in protein expression at both 3 and 7 days after WBIR (**Figures 2B, C**) compared to sham mice. Three days after 2 Gy exposure, 508 proteins were significantly increased, and 80 proteins were reduced (**Figure 2B**) compared to sham mice. Similarly, 7 days after 2 Gy of WBIR, there were 408 upregulated proteins, and 80 downregulated proteins in EVs (**Figure 2C**). In order to identify canonical pathways inferred from the proteomic analysis, we employed Ingenuity Pathway Analysis (IPA) with resulting Z-scores reflecting increased or decreased numbers of gene members of each canonical pathway compared to sham mice. IPA predicted several protein categories were altered by 2 Gy, with similar changes at days 3 and 7 (**Supplemental Figures 1A, B**). Interestingly, the most impacted categories are often observed as being disrupted in tissues after WBIR, such as intracellular and second messenger signaling, cell proliferation and growth, cellular stress and injury, apoptosis, and cellular immune responses (**Supplemental Figures 1A, B**) (5). Proteins involved in integrin signaling, actin cytoskeletal, and RHOGDI signaling proteins were notably altered, as were proteins associated with phagosome formation. Next, we performed analysis of a higher WBIR dose to test the hypothesis that EV-bound protein changes will reflect the WBIR dose received. Indeed, EVs isolated after 9 Gy exposure had significantly different changes in protein cargo compared to EVs isolated at 2 Gy. This was the case at both timepoints after exposure (**Figures 2B–E**). After the 9 Gy exposure, EV protein cargo changed quite differently when compared to 2 Gy, with fewer changed proteins compared to the 2 Gy treatment group. Three days after 9 Gy of WBIR, only 94 proteins were significantly increased, and 72 proteins significantly decreased (**Figure 2D**). Seven days after 9 Gy WBIR, 148 proteins were significantly up-regulated and 98 proteins were down-regulated (**Figure 2E**) compared to sham mice. IPA analysis found significant increases in proteins related to estrogen receptor signaling and the complement system.

In order to refine the potential use of EV-bound proteins as biomarkers, we performed several further analyses. First, we assessed specific EV-bound proteins that were altered over time at the same WBIR dose. We found that 7 days after 2 Gy exposure, there were 100 proteins increased and 173 proteins decreased compared to day 3 (**Figure 2F**). IPA identified that these protein changes were associated with RHOA signaling, actin cytoskeleton signaling, VEGF, and glycolysis, while there were fewer proteins involved in the intrinsic prothrombin pathway, calcium transport, SNARE signaling, and glycoprotein 6 signaling (**Figure 2G**). After 9 Gy exposure, only 12 proteins significantly changed between days 7 and 3 (**Figure 2H**). Although there were not enough significantly altered proteins for IPA to assign a Z-score, we found that these proteins were involved in several pathways, notably LXR/RXR signaling, cytoskeleton-related pathways, and phagosome formation (**Figure 2I**). In an effort to identify proteins that might serve as biomarkers of exposure, we assessed the similarity in changes across both doses and timepoints (**Figure 2J**). Across all the timepoints and doses, 65 shared protein changes were found with 35 increased and 30 were reduced (**Figure 2J** red square, **Tables 1**, **2**). Among these, thromboxane-A synthase (~10-fold), fibrinogen alpha and gamma chains (~7-8-fold), and lymphocyte cytosolic protein 2 (lcp2, ~6-fold) were the most increased (**Table 1**). The increase in lcp2 was also seen by ELISA (**Supplemental Figure 1E**). Among the shared reduced proteins, vasorin and alpha-1-antitrpysin 1-5 showed the greatest reduction in EVs after WBIR (**Table 2**). To identify potential biomarkers of high doses of radiation exposure that could be used at different times after exposure, we compared the protein changes at 3 and 7 days following 9 Gy WBIR. Twelve proteins overlapped (**Figure 2J**, red circle). Notably, suprabasin and sarcalumenin were increased, whilst cathelicidin antimicrobial peptide (Camp) and copine-1 showed the greatest decrease (**Table 3**).

**Table 1:** Significantly up-regulated proteins across all doses and timepoints.

Gene Name	Protein Description	Log2 Fold Change 2 Gy Day 3	Log2 Fold Change 2 Gy Day 7	Log2 Fold Change 9 Gy Day 3	Log2 Fold Change 9 Gy Day 7
Tbxas1	Thromboxane-A synthase	10.34	9.14	10.07	9.96
Fga	Fibrinogen alpha chain	8.05	6.80	7.91	7.82
Fgg	Fibrinogen gamma chain	7.38	6.30	7.39	7.23
Lcp2	Lymphocyte cytosolic protein 2	6.59	5.82	6.65	6.50
Fgb	Fibrinogen beta chain	6.67	5.61	6.68	6.48
Hsp90aa1	Heat shock protein HSP 90-alpha	6.20	5.54	6.29	6.18
P4hb	Protein disulfide-isomerase	6.29	5.18	4.63	3.59
Cyb5a	Cytochrome b5	6.69	3.98	4.08	3.05
Cavin1	Caveolae-associated protein 1	5.27	3.87	4.17	3.92
Esyt1	Extended synaptotagmin-1	5.71	4.01	3.43	3.43
Ubash3b	Ubiquitin-associated and SH3 domain-containing protein B	3.10	5.63	3.71	3.67
Actn1	Alpha-actinin-1	3.59	6.02	1.74	4.25
Tmed10	Transmembrane emp24 domain-containing protein 10	5.94	3.44	3.65	2.24
Cd81	CD81 antigen	5.00	3.91	3.67	2.51
Pon3	Serum paraoxonase/lactonase 3	5.48	2.57	3.30	3.61
Cyb5r3	NADH-cytochrome b5 reductase 3	5.30	3.68	3.30	2.63
Tapbp	Tapasin	5.59	3.78	2.96	2.22
Aadacl4	Arylacetamide deacetylase-like 4	4.94	2.44	3.32	3.75
Hsp90b1	Endoplasmin	4.15	4.28	3.33	2.10
Pdia3	Protein disulfide-isomerase A3	4.61	3.55	3.27	2.16
Clic1	Chloride intracellular channel protein 1	3.33	4.31	2.25	2.79
Mvp	Major vault protein	4.87	2.91	3.13	1.75
Atp2a1	Sarcoplasmic/endoplasmic reticulum calcium ATPase 1	3.94	2.62	2.50	3.48
Gp1bb	Platelet glycoprotein Ib beta chain	4.08	3.71	1.82	2.73
Atp1a2	Sodium/potassium-transporting ATPase subunit alpha-2	3.10	2.57	2.76	3.33
Mmp2	72 kDa type IV collagenase	2.21	2.65	3.38	3.25
Thsd4	Thrombospondin type-1 domain-containing protein 4	5.60	2.23	2.69	0.69
F13a1	Coagulation factor XIII A chain	3.06	1.96	2.89	1.79
Rps6	40S ribosomal protein S6	2.85	1.76	2.70	1.92
Ca1	Carbonic anhydrase 1	2.00	2.42	2.33	1.76
Gapdh	Glyceraldehyde-3-phosphate dehydrogenase	1.81	1.44	1.94	1.53
Vamp8	Vesicle-associated membrane protein 8	1.98	1.26	1.29	1.00
F13b	Coagulation factor XIII B chain	1.66	0.91	1.78	1.10
Pros1	Vitamin K-dependent protein S	1.84	0.88	1.24	1.36

**Table 2:** Significantly down-regulated proteins across all doses and timepoints.

Gene Name	Protein Description	Log2 Fold Change 2 Gy Day 3	Log2 Fold Change 2 Gy Day 7	Log2 Fold Change 9 Gy Day 3	Log2 Fold Change 9 Gy Day 7
Vasn	Vasorin	-3.78	-5.05	-4.28	-5.53
Serpina1e	Alpha-1-antitrypsin 1-5	-2.87	-4.83	-4.21	-3.63
Gda	Guanine deaminase	-2.75	-3.54	-3.29	-3.97
Prg4	Proteoglycan 4	-2.99	-3.88	-3.30	-3.21
C1sb	Complement C1s-B subcomponent	-3.02	-3.61	-3.20	-3.20
Ttr	Transthyretin	-4.41	-2.08	-2.54	-2.14
Capn1	Calpain-1 catalytic subunit	-1.53	-2.82	-2.46	-2.81
Serpina1d	Alpha-1-antitrypsin 1-4	-2.27	-2.25	-2.49	-2.29
Serpina3k	Serine protease inhibitor A3K	-2.10	-2.52	-2.22	-2.07
C1sa	Complement C1s-A subcomponent	-1.94	-2.64	-2.16	-2.11
Hspa9	Stress-70 protein, mitochondrial	-2.42	-1.12	-2.91	-2.08
Serpina1a	Alpha-1-antitrypsin 1-1	-1.90	-2.16	-2.28	-2.16
C5	Complement C5	-2.25	-2.24	-1.64	-1.80
C1ra	Complement C1r-A subcomponent	-1.56	-2.75	-1.91	-1.69
A2m	Alpha-2-macroglobulin-P	-2.48	-1.13	-1.90	-2.23
Park7	Parkinson disease protein 7 homolog	-2.84	-0.84	-1.88	-2.17
Serpina3n	Serine protease inhibitor A3N	-2.74	-2.47	-1.10	-1.27
Serpina3m	Serine protease inhibitor A3M	-2.04	-2.02	-1.72	-1.73
Bdh1	D-beta-hydroxybutyrate dehydrogenase	-1.40	-1.65	-1.49	-1.43
Spp2	Secreted phosphoprotein 24	-1.42	-1.44	-1.72	-1.34
Masp2	Mannan-binding lectin serine protease 2	-1.28	-1.42	-1.26	-1.56
Psma1	Proteasome subunit alpha type-1	-1.16	-0.71	-1.29	-2.05
Serpina1b	Alpha-1-antitrypsin 1-2	-1.02	-1.22	-1.34	-1.18
Gpx3	Glutathione peroxidase 3	-1.32	-0.85	-0.86	-1.30
Ica	Inhibitor of carbonic anhydrase	-1.35	-0.77	-0.78	-0.72
Cfp	Properdin	-0.74	-0.92	-0.75	-1.09
Ifnar2	Interferon alpha/beta receptor 2	-0.71	-1.10	-0.84	-0.73
Prdx2	Peroxiredoxin-2	-0.56	-0.71	-0.92	-1.12
Atp5f1e	ATP synthase subunit epsilon	-0.91	-0.76	-0.77	-0.66
Azgp1	Zinc-alpha-2-glycoprotein	-1.03	-0.66	-0.51	-0.60

**Table 3:** Significantly altered EV proteins after 9 Gy WBIR.

Gene Name	Protein Description	log2 Fold Change day 3	log2 Fold Change day 7
Sbsn	Suprabasin	2.16	3.65
Srl	Sarcalumenin	1.95	2.53
Plxna4	Plexin-A4	-1.41	-1.72
Pf4	Platelet factor 4	-1.51	-2.31
Pdcd6	Programmed cell death protein 6	-2.44	-1.76
Vcam1	Vascular cell adhesion protein 1	-1.93	-1.72
Cat	Catalase	-1.91	-2.36
Aadac	Arylacetamide deacetylase	-2.13	-2.35
Steap3	Metalloreductase STEAP3	-4.23	-2.19
Dnpep	Aspartyl aminopeptidase	-2.45	-3.54
Cpne1	Copine-1	-3.59	-4.05
Camp	Cathelicidin antimicrobial peptide	-4.27	-5.62

Beyond protein changes, we and other groups have also demonstrated that EV-bound miRNA can also serve as potential biomarkers (38, 46). Therefore, we assessed alterations in the miRNA content of EVs using nanoString’s nCounter mouse miRNA panel. Three days after injury there were 15 miRNAs that changed similarly at both doses (**Figure 2K**). Notably, miR-376a and miR-136 were increased over 100 to 200-fold and 30 to 60-fold at 2 Gy and 9 Gy respectively. Further there were 7 miRNAs specific to the high dose 9 Gy, exposure (**Figure 2L**). Taken together, these unbiased assessments of EV cargo have identified proteins and miRNAs that could serve as biomarkers for radiation exposure, and they may also act as possible drivers of the immune and physiologic dysfunction observed during ARS.

### WBIR-induced EVs induce immune dysfunction consistent with exposure to radiation

**Figure 3:**
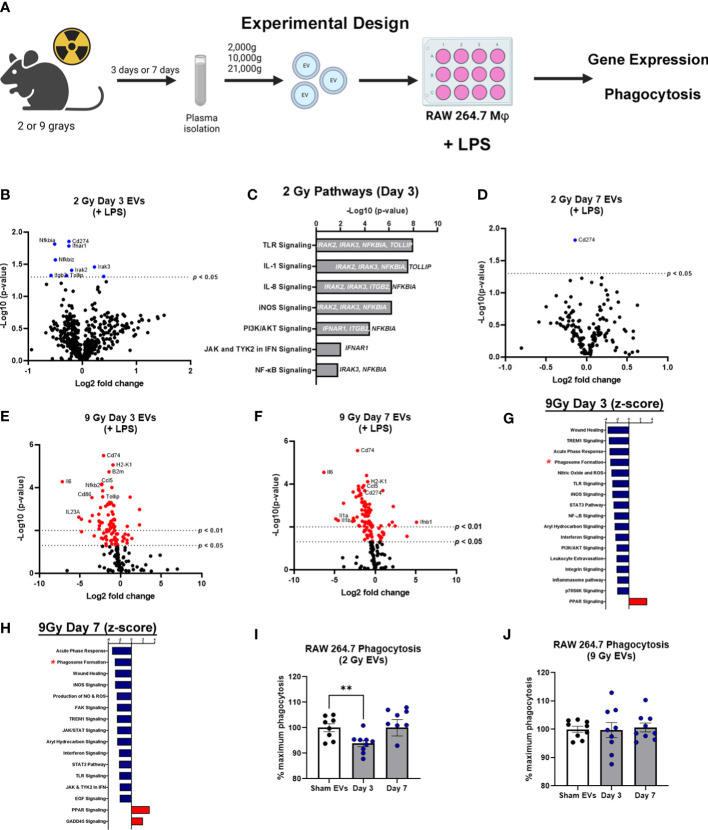
EVs released following WBIR induce immune gene expression changes that reflect exposure to radiation. **(A)** Experimental overview for the *in vitro* exposures and analyses involving WBIR-induced EVs. **(B)** Volcano plots displaying the immune gene changes in RAW macrophages exposed to EVs isolated 3 days after 2 Gy of WBIR compared to Sham EVs in the presence of LPS (n=3 for each experimental group). **(C)** IPA analysis of the pathways that were determined to be most impacted. **(D)** Volcano plots displaying the immune gene changes in RAW macrophages exposed to EVs isolated 7 days after 2 Gy of WBIR compared to Sham EVs in the presence of LPS (n=3 for each experimental group). **(E, F)** Volcano plots displaying the immune gene changes in RAW macrophages exposed to EVs isolated **(E)** 3 days or **(F)** 7 days after 9 Gy of WBIR compared to Sham EVs in the presence of LPS (n=3 for each experimental group). Canonical immune pathways identified to be most impacted by IPA with their associated Z-scores after **(G)** 3 days or **(H)** 7 days. **(I, J)** Quantification of the phagocytic capability of macrophages during co-culture of EVs released after 2 Gy **(I)** and 9 Gy **(J)** of WBIR (n=8-9 for each experimental group); average fluorescence units of no-cell negative-control wells from all wells. We then defined phagocytosis response to the experimental effector (% Effect) as: % Effect = Net experimental phagocytosis × 100% x Net positive control phagocytosis. *p<0.05, One-way ANOVA with Dunnett’s *post-hoc* test. Created with BioRender.com.

EVs have emerged as potential contributors to the bystander effect that is observed following exposure to radiation (27). Therefore, to determine the effect of EVs on immune dysfunction/immune reprogramming after WBIR, EVs were isolated after exposure (3 or 7 days) to WBIR (2 or 9 Gy). WBIR-EVs were then administered to RAW 264.7 macrophages treated with LPS using our *ex-vivo* protocols previously used in the setting of burn injury (35, 36). Immune gene expression was measured 24 hours after exposure using nanoString technology (**Figure 3A**) which allows for the simultaneous quantification of 561 immune genes. WBIR-EVs blunted immune responses to LPS, with the most severe impact seen with 9 Gy exposure. EVs isolated 3 days after 2 Gy exposure resulted in a significant down-regulation in seven immunoregulatory genes: *NFκBia*, *NFκBiz CD274*, *Ifnar1, Itgb2, Tollip, and Irak2* (**Figure 3B**), compared to sham EVs. Although there were not enough significantly altered genes for IPA to assign Z-scores, we found that 3 day WBIR-induced EVs, compared to sham-EV, induced significant changes in key regulatory genes with an overall predicted increase in pro-inflammatory TLR, IL-1 and iNOS signaling with a corresponding increase in NFκB signaling (**Figure 3C**). These changes are consistent with the hyper-inflammatory response associated with ARS). EVs isolated 7 days after 2 Gy, however, showed almost no significant changes with only a slight reduction in *CD274* (**Figure 3D**) compared to sham EVs. This return to baseline by day 7 is possibly consistent with the survivability of this level of exposure. In contrast, EVs isolated 3 (**Figure 3E**) and 7 days (**Figure 3F**) after 9 Gy exposure showed profound suppression of macrophage responses to LPS, with IPA predicting inhibition of wound healing, inflammatory signaling, leukocyte extravasation, nitric oxide production, aryl hydrocarbon receptor signaling, and phagosome formation (**Figures 3G, H**). PPAR signaling, however was predicted to be activated after exposure to EVs from all time points compared to sham EVs.

Since IPA predicted an inhibition of phagosome formation, and we have previously shown that EVs released after burn injury also influence the phagocytic capability of macrophages (35), we assessed the effect of WBIR-EVs on phagocytosis. RAW macrophages were co-cultured with LPS, fluorescein-labelled killed *E. coli* (K-12 Stain), and WBIR-Induced EVs or Sham EVs. EVs isolated 3 days after 2 Gy exposure significantly inhibited phagocytosis compared to sham EVs; however 2 Gy EVs isolated at 7 days had no impact on phagocytosis compared to sham EVs (**Figure 3I**). EVs induced by 9 Gy exposure did not result in a significant reduction in phagocytosis when isolated both 3 and 7 days after injury (**Figure 3J**). Since these data indicates that EVs may contribute to ARS-associated immune dysfunction, we next investigated if MSC-EVs, shown in many systems to restore overt immune reprogramming to homeostasis, could be used to reduce the immune dysfunction associated with ARS.

### MSC-EVs restore immune function to homeostasis when given after either WBIR or radiation combined with burn injury

**Figure 4:**
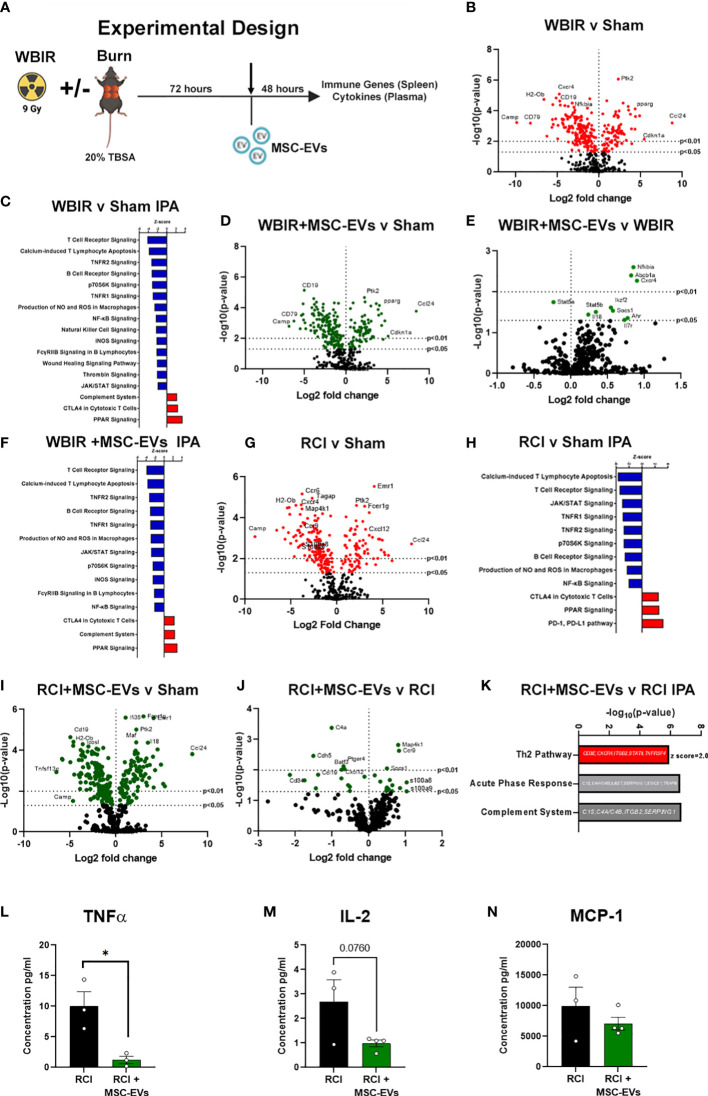
MSC-EVs restore immune function to homeostasis when given after either WBIR or radiation combined with burn injury (RCI). **(A)** Experimental design displaying the models of both WBIR and RCI and the treatment with MSC-EVs 3 days after injury before the harvesting of spleens and cytokines. **(B)** Volcano plot displaying the immune gene changes and **(C)** IPA pathways that were most impacted following exposure to WBIR (n=3 for each experimental group). **(D, E)** Volcano plots displaying gene expression changes in mice treated with MSC-EVs following exposure to WBIR compared to **(D)** sham and **(E)** untreated WBIR mice (n=3 for each experimental group). **(F)** IPA analysis displaying the most significantly impacted pathways with MSC-EV treatment compared to sham mice. **(G)** Volcano plots illustrating immune gene alterations in mice exposed RCI compared to sham mice and the **(H)** IPA analysis of these immune pathways. **(I, J)** Volcano plots demonstrating the impact of MSC-EVs on immune gene changes following **(I)** RCI compared to sham mice **(J)** vs RCI alone mice. **(K)** IPA analysis of RCI+MSC-EVs vs RCI alone. The Th2 pathway was increased by MSC-EVs. Acute phase response and complement showed gene changes depicted. **(L–N)** Plasma cytokine levels of **(L)** TNFα, **(M)** IL-2, and **(N)** MCP-1 mice treated with MSC-EVs following RCI (n=3-4 for each experimental group). *p<0.05, One-way ANOVA with Dunnett’s *post-hoc* test. Created with BioRender.com.

To determine if MSC-EVs can act as a putative MCM to restore ARS-induced immune function to one closer to health, we utilized the WBIR model plus a model of polytrauma, RCI, in which we have observed a greater amplitude of immune dysfunction than WBIR alone (**Figure 4A**). Mouse MSC-EVs were purified from MSCs and isolated by differential centrifugation. 1x10^10^ MSC-EV/mouse resuspended in sterile saline, or sterile saline alone, were injected *via* tail vein into mice 72 hours after WBIR, RCI or sham injury. Forty-eight hours later, mice were sacrificed, and we harvested the spleen to assess the transcription of immune genes using nanoString, and plasma for quantification of peripheral immune cytokines and chemokines by Bio-plex multiplex assay (**Figure 4A**). Firstly, to demonstrate the specific immune gene reprogramming associated with WBIR versus sham injury, in the absence of MSC-EV treatment, nanoString analysis revealed that WBIR caused profound alterations in peripheral immune gene expression with 264 genes significantly altered (**Figure 4B**, log_2_FC range -10 to 10) compared to sham mice. Of note, *Camp* was significantly reduced (log_2_FC= -9.8) by WBIR and was also the most significantly reduced protein in EVs after WBIR (**Table 3**). IPA revealed a downregulation of several key immune pathways related to ARS (**Figure 4C**), *versus* sham injured mice, with many similarities to the IPA of RAW cells exposed to 9 Gy WBIR-induced EV (**Figure 3C**). Particularly, an “immunosuppressed phenotype” was observed with reduced T cell signaling, TNF and NFκB, as well as wound healing and significantly increased PPAR signaling (**Figures 3G–H**). The immune checkpoint inhibitor CTLA4 pathway was also increased by WBIR, compared to sham mice, congruent with the reductions in T cell signaling.

We then investigated the effect of MSC-EV treatment on the peripheral immune responses after sham or WBIR injury. Firstly, we compared MSC-EV-treated WBIR mice to untreated sham-injured mice. Utilizing splenic mRNA, we found that MSC-EV treatment normalized certain key WBIR-induced immune gene changes with 245 total genes changing (**Figure 4D**), compared to untreated sham injured mice (contrast these data with the 264 genes altered in untreated WBIR mice versus sham mice, **Figure 4B**). Therefore, to complete this analysis, we also examined the effect of MSC-EV treatment of WBIR injured mice compared to untreated WBIR injured mice (**Figure 4E**). This comparison directly identified the key genes which were altered specifically by MSC-EV treatment in injured mice (e.g. *NFκBia, Cxcr4, Socs1* were significantly upregulated, and *Stat5a* was significantly downregulated) *versus* untreated mice. In addition, MSC-EV treatment increased aryl hydrocarbon signaling which was shown to be inhibited in RAW macrophages exposed to EVs isolated following exposure to 9 Gy of WBIR (**Figures 3G, H**, **4E**). Taken together, these data suggest the majority of genes were not significantly altered by MSC-EVs, however, there were specific genes returned to homeostasis. These findings were reflected in the IPA of the MSC-EV treated *versus* untreated WBIR-injured mice (**Figure 4F**), with immune signaling pathways mainly remaining unchanged. Comparing this analysis with the specific immune gene reprogramming associated with WBIR versus sham injury (**Figure 4F** *versus* **Figure 4C**), it is clear that most WBIR-dependent signaling changes, such as downregulation of T cell Receptor, NFκB and upregulated PPAR signaling were not altered by MSC-EV treatment. However, the wound healing pathway, thrombin signaling were not reduced when MSC-EVs were given, and a downregulation of the P70s6K and “FCγRIIB signaling in B lymphocytes” pathways occurred with MSC-EV treatment of WBIR injury compared to untreated WBIR mice.

Turning next to our RCI model, we observed that RCI *versus* sham injury caused similar widespread immune gene changes with 233 genes changing (**Figure 4G**) and similar pathway disturbances (**Figure 4H**), both comparable to the gene and pathway changes induced with WBIR (**Figures 4B, C**). Also similar to WBIR, we found that MSC-EV treatment normalized certain key RCI-induced immune gene changes with 240 total genes changing (**Figure 4I**) and minimal changes to immune signaling pathways (not shown) compared to untreated sham injured mice (compare **Figure 4I** with **Figure 4G**). When we treated RCI mice with MSC-EV and compared the splenic immune gene expression with untreated RCI-mice, we found a slight yet significant reprogramming of the immune response (**Figure 4J**), with more genes impacted than in the WBIR model (26 genes compared to 10 in the WBIR model). In comparison to the untreated RCI response (**Figure 4G**) certain key immune regulatory genes reduced by RCI were improved by MSC-EVs such as *Map4k1*, *Ccr9*, *s100a8/9*, and *Cxcl12.* IPA of these data revealed a significant re-programming of the immune signaling pathways in MSC-EV treated mice compared to untreated mice (**Figure 4K**), with a shift towards a regenerative anti-inflammatory Th2 responses and a reduction in the highly inflammatory Acute Phase Response pathways, so intrinsically involved with the induction of the hyper-inflammation associated with both ARS and burn injury.

We then tested whether these gene changes translated into differences in functional protein expression. Multiplex cytokine and chemokine analysis of the plasma harvested from RCI-mice after MSC-EV treatment or treatment revealed a significant reduction in TNFα protein levels after RCI with MSC-EVs (**Figure 4L**), a trend toward a reduction in IL-2 (**Figure 4M, N**). MCP-1 did not approach statistical significance (*p=*0.36; **Figure 4N**). These findings are consistent with partial normalization of immune changes by MSC-EVs given 3 days after RCI injury. Taken together, these data demonstrate in two different pre-clinical models of radiation injury that MSC-EV may act to normalize the profound immune dysfunction associated with ARS.

### MSC-EVs can significantly improve survival if given before WBIR exposure

**Figure 5:**
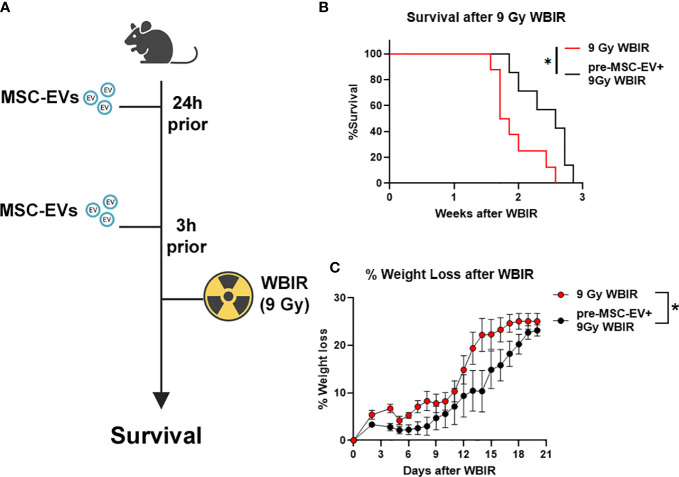
MSC-EVs act as a radioprotectant if given before exposure to 9 Gy of WBIR. **(A)** Experimental design for assessing the efficacy of MSC-EV pre-treatment for protecting against WBIR. **(B)** MSC-EV pre-treatment prevents mortality and limits **(C)** weight loss following exposure to a lethal dose of WBIR (n=7-8 for each experimental group). *p<0.05, One-way ANOVA with Dunnett’s *post-hoc* test. Significant interaction between time after radiation and treatment was assessed using a Mixed-effects model (REML), F_19,234 = _2.105, *p=*0.0055. Created with BioRender.com.

The *in vivo* stability of EVs combined with the homeostatic effects of EVs as described above suggest that they may act as potent radioprotectants with long clearance time. To the best of our knowledge, there have not been any studies evaluating if MSC-EVs can act as a radioprotectant if given prophylactically before lethal doses of WBIR. Therefore, we treated mice with MSC-EVs (i.v, 10^10^/mouse in saline) twice prior to exposure (24 and 3 hrs) to 9 Gy WBIR (**Figure 5A**). The 9 Gy exposure was fatal with all mice dying within 3 weeks. Pre-treatment with MSC-EVs significantly prolonged survival (**Figure 5C**, 1.8 *versus* 2.6 weeks, 9 Gy *versus* 9G+MSC-EVs). MSC-EV pretreatment accordingly slowed the progression of weight loss after exposure (**Figure 5B**). Thus, MSC-EV pretreatment can slow the progression of mortality after WBIR injury.

## Discussion

**Figure 6:**
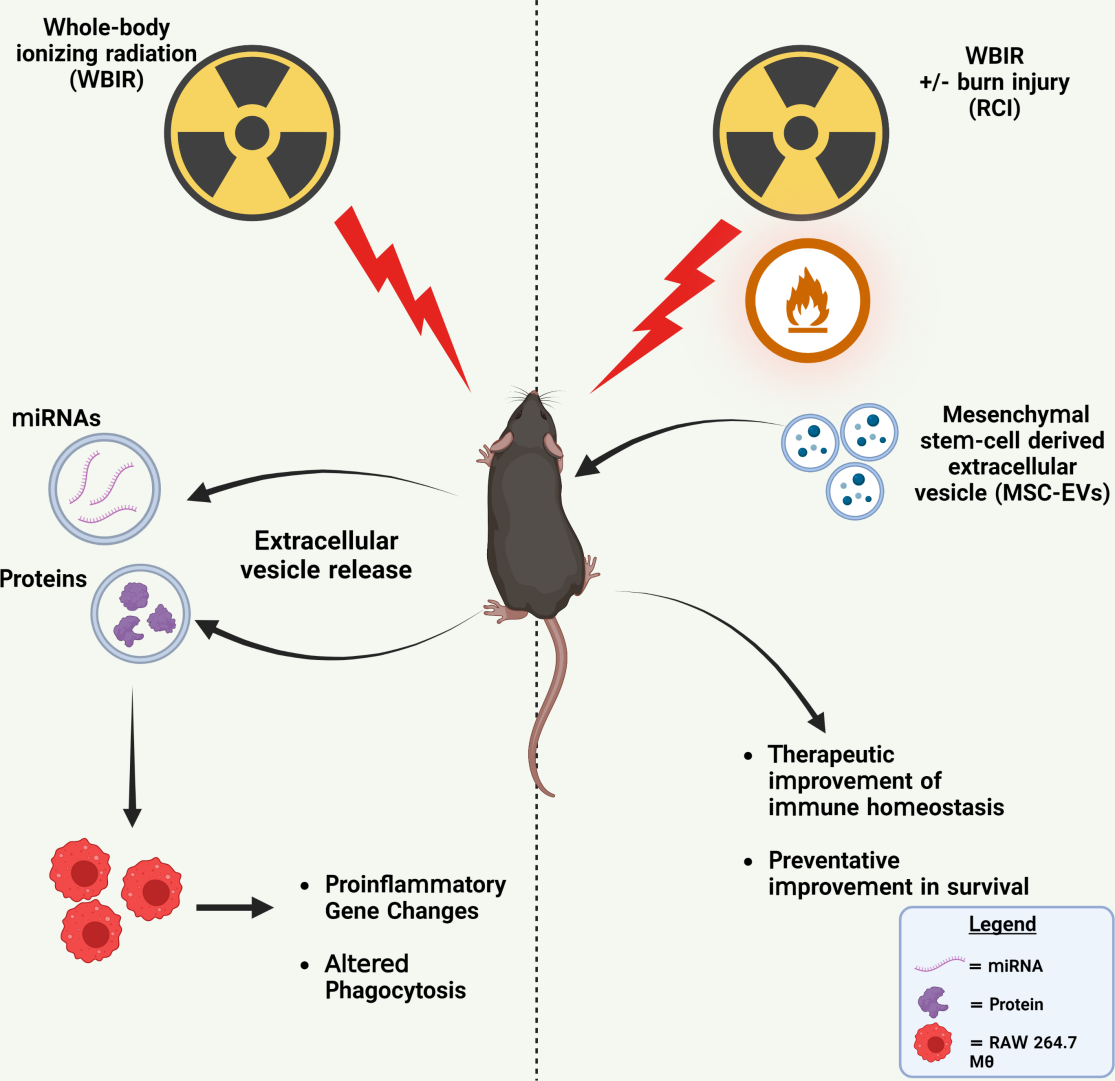
Summary of findings. Left: WBIR causes alterations in miRNA and protein cargo of circulating EVs that suppressed proinflammatory immune gene induction and phagocytic responses to LPS in RAW 264.7 macrophages. Right: The preventative and therapeutic administration of MSC-EVs with either WBIR or RCI protected against WBIR exposure and improved immune homeostasis. Created with BioRender.com.

Due to the heightened threats of nuclear warfare, terrorist attacks, or nuclear power plant accidents, there is an increasing likelihood of a radiological incident occurring within the general population. Because of the severity of these scenarios, it is likely that clinicians may have to treat thousands of patients that were exposed to high doses of radiation (47). Currently, there are not any effective bio-dosimetry markers for predicting the radiation dose received and very few therapeutics for treating ARS. In this study, we demonstrate that EVs released after exposure to WBIR have altered proteomic and miRNA cargo that is related to the dose of radiation received and time after exposure. This data could be utilized to assist in triaging and the clinical treatment of patients. Furthermore, we demonstrate that these EVs induce immune gene changes in cultured monocytes which dampened pro-inflammatory signaling and inhibited phagocytosis. We also assessed if treatment with MSC-EVs could be used as an effective therapeutic for treating both WBIR and RCI. We found that MSC-EVs slightly improved immune homeostasis following both WBIR and RCI and reduced pro-inflammatory cytokine levels after RCI. Further, we found that MSC-EVs could act as a radioprotectant if given prior to WBIR. Taken together, this data demonstrates that EVs contain cargo that promotes radiation-induced immune effects and that they could be utilized as a potential bio-dosimetry marker. Prophylactic or therapeutic treatment with MSC-EVs limits the harmful effects of exposure to WBIR (**Figure 6**).

Following exposure to WBIR, we found there was a significant reduction in circulating plasma EV levels 3 days after exposure to 9 Gys (**Figure 1A**). However, there were not any significant differences in circulating EV levels across the other timepoints and doses. Other conditions associated with tissue injury such as trauma and burn injury have been typically associated with an increase in the number of plasma EVs, we were surprised to observe only a transient reduction of plasma EVs after 9 Gy of WBIR (36, 42). This could be due to the massive cell death seen after 9 Gy of WBIR or a transient cellular shock caused by the injury resulting in the shedding of fewer EVs. This has been similarly observed in cancer cells exposed to 9 Gy of gamma irradiation which resulted in dramatic decreases in EV secretion (48). We also found that there were alterations in EV proteomic and miRNA cargo that reflect the dose of WBIR received and time after exposure. Interestingly, the more dramatic alterations in the EV protein cargo occurred following exposure to 2 Gy while the protein changes for the 9 Gy dose were much less pronounced (**Figures 2B–E**). However, there was a significant reduction in the total number of EVs 3 days after exposure to 9 Gy of WBIR and no significant changes in the total number of EVs 3 days after 2 Gy of WBIR. It is important to note that exposure to 2 Gy of WBIR for a mouse is not lethal and mice are able to recover from this exposure while a 9 Gy dose of WBIR is lethal and the mice are unable to recover (8). Thus, the lower number of circulating EVs three days after 9 Gy could be more detrimental than the lack of proteomic changes. Based on the proteomic changes following exposure to 2 Gy of WBIR, IPA identified a plethora of pathways that were impacted (**Supplemental Figures 1A, B**). These changes may represent a functional adaptation during recovery from 2 Gy that does not occur with the larger 9 Gy dose due to massive cell death and fewer healthy cells secreting EVs into the periphery. When comparing the temporal proteomic changes between day 7 and day 3 following exposure to 2 Gy, some of the notable pathways that were activated were related to Rhoa and actin cytoskeleton signaling (**Figure 2G**). Consistent with this finding, previous studies has found that exposure to ionizing radiation in melanoma leads to actin rearrangements and the thickening of actin fibers which may be mediated by Rhoa signaling (49, 50). Based on these protein changes in our EV cargo, it is possible that EVs could be mediating these effects of ionizing radiation. Further, there was a down-regulation in pathways associated with prothrombin and glycoprotein-6 signaling, a key platelet pathway, 7 days after exposure to 2 Gy of WBIR (**Figure 2G**). Bleeding diathesis is one of the most common symptoms associated with radiation exposure (51) and an inhibition of these pathways by EVs could be mediating these symptoms.

Regarding the use of EVs as biomarkers of exposure, there were 65 proteins that were significantly altered across all the doses and timepoints (**Tables 1**, **2**). Among the proteins that had the highest fold-change across all groups were thromboxane A synthase and lymphocyte cytosolic protein 2 which play essential roles in platelet function and T-cell receptor signaling, respectively (52, 53). Among proteins decreased across all groups, the transforming growth factor, vasorin, was significantly decreased (54). For the 9 Gy dose there were only 12 proteins that were significantly altered between timepoints and suprabasin and sarcalumenin were increased, while Camp and copine-1 were decreased (**Table 3**). Based on these findings, a panel for detecting increases of EV proteins that were increased among all groups (e.g. thromboxane A synthase and lymphocyte cytosolic protein 2) could be used to identify if there was any radiation exposure, while the increases of proteins in the 9 Gy group (suprabasin and sarcalumenin) might be useful as an indicator for a high dose of radiation. Interestingly, Camp was significantly decreased in the EV protein cargo while the *Camp* gene was also found to be down-regulated in spleens of irradiated mice (**Table 3**, **Figure 4B**). Beyond proteins, EVs are rich in miRNAs. Therefore, we assessed if there were changes in the miRNA cargo of these EVs following WBIR exposure.

Between both the 2 Gy and 9 Gy dose, there were 15 significantly altered EV-bound miRNAs across both groups (**Figure 3K**). In addition, the directionality of these miRNAs was similar between both the 2 and 9 Gy doses. With both doses, there was an enormous fold-change increase in miR-136 and miR-376a. The fold changes in these miRNAs were slightly more dramatic in the 9 Gy dose as expected. For instance, miR-136 was increased 30-fold in EVs isolated following 2 Gy, while it was increased 63-fold in EVs after 9 Gy. In cancer, miR-136 has been shown to promote apoptosis and actively represses anti-apoptotic genes (55). Direct exposure to ionizing radiation causes the mass apoptosis of numerous different cell types and can be mediated by the bystander effect (27). It is possible that the increase in miR-136 in EVs after WBIR could be involved in these effects. Prior work has found that miR-376a sensitizes cells to DNA damage, making them unable to repair DNA breaks which causes genomic instability (56). Since exposure to ionizing radiation causes DNA damage, an up-regulation in this miRNA may indicate that these EVs are exacerbating some of the effects of exposure to ionizing radiation. Future studies will investigate the role of these miRNAs in post-WBIR cell death and DNA damage. In addition, there were seven miRNAs that were significantly changed in the 9 Gy dose alone (**Figure 3L**). Based on this data, we propose that these EV-bound miRNA changes could be used as biomarkers for detecting radiation dose. For instance, the detection of miR-136 and miR-376a in EVs could be used to identify if there was any exposure to radiation, while the identification of miRNAs specific to the 9 Gy dose could indicate that there was an exposure to a high-dose of radiation. Others have identified a significant down-regulation in EV bound miRNA 142 after 2 Gy of WBIR which we also observed (18). Experiments are underway to further identify classes of biomarkers and a multiparametric bio-dosimetry algorithm(s) with the potential to detect IR exposure, time since exposure and/or exposure dose. Also, outside of miRNAs, EVs are also rich in IncRNAs and CircRNAs and future experiments will explore if there are alterations in these RNA species (57). The feasibility of use in non-invasive (e.g., use of salivary EV) screening for radiation exposure will then be explored. Since these alterations in EV cargo were shown to impact numerous pathways (**Supplemental 1A–D**), we next performed *in vitro* exposures to evaluate if WBIR-induced EVs influenced immune gene expression and functional alterations.

Previous work in our lab and others have demonstrated that macrophages play an important role in immune dysfunction following severe burn injuries (35, 36). Macrophages are also important following radiation exposure, where they are responsible for the removal of apoptotic cells and elicit phagocytic functions (58). EVs isolated 3 and 7 days after 9 Gy of WBIR robustly reduced the macrophage immune transcriptome response to LPS, suggesting these EVs may physiologically dampen macrophage responses in the setting of ARS. These immune gene changes and IPA analysis were remarkably similar between macrophages exposed to EVs isolated 3 or 7 days after exposure to 9 Gy (**Figures 3E–H**). Interestingly, while we observed the most dramatic protein changes in the EVs released after 2 Gy, only seven immune genes were blunted in the macrophage response to LPS. By day 7, the influence of these EVs on gene expression were essentially gone, with *Cd74* being the only gene that was significantly different. A limitation of this study is that we only analyzed transformed macrophage immune transcriptome responses to EV, and therefore these data can only be extrapolated to physiological responses in primary immune cells. We are currently testing if these transcriptomic responses do indeed translate to *in vivo* immune functional alterations.

Since the 2 Gy dose of radiation is survivable, this is consistent with a return to homeostasis by day 7. This was also observed with the phagocytosis assay. EVs isolated 3 days after 2 Gy inhibited phagocytosis whereas EVs isolated 7 days after 2 Gy did not (**Figure 3I**). While we only looked at phagocytosis in this study, future studies should evaluate if the inhibition of these pathways also correlate with other functional assays. For instance, assessing if these EVs released after WBIR limit healing of damaged tissue or promote barrier dysfunction, as the IPA predicted. While WBIR-induced EVs can cause immune dysfunction, we found that MSC-EVs could serve as therapeutic that could reverse or prevent these effects.

MSC-EVs have emerged as potent immunomodulatory molecules that are anti-inflammatory, promote wound healing, and regeneration (59). There are no FDA-approved treatments for the clinical outcomes of ARS after high dose IR-exposure. While stem cell therapy has been used to develop radiation MCM, studies have suggested that the secretome of these stem cells contained the critical growth factors and signaling molecules for the stem cell-driven regeneration *via* EVs. (60). In the context of radiation exposure, MSC-EVs have been shown to mitigate intestinal and hematopoietic damage when given after exposure to WBIR (28, 29). In addition, MSC-EVs are a promising therapeutic for the treatment of these forms of injury because they can be produced on a mass scale, lack histocompatibility complexes (i.e. low risk of donor incompatibility), and can be administered rapidly in response to emergencies (61). We found that MSC-EVs given 3 days after WBIR slightly and selectively restore immune homeostasis after RCI and WBIR. For instance, *Cish1* and *Socs1* (both members of the Suppressors of Cytokine Signaling (SOCS) family, involved with negative regulation of cytokine inhibition) and *Nfκbia* (NFκB Inhibitor Alpha) were upregulated in IR-injured mice following MSC-EV treatment, while *Stat5a* is significantly downregulated. These data are interesting as they are consistent with data from other animal models which also showed that *Cish1*, *Socs* and *Nfκbia* are associated with restoration of immune homeostasis and were slowly upregulated after long periods (weeks) during recovery from IR exposure (62). We also show a treatment-dependent increase in PPARγ gene expression and PPARγ signaling pathways, which reside at the intersection of immune and metabolic pathways (63–65); PPARγ is a negative regulator of mTOR which is activated after TLR/MyD88 engagement. Reduction in mTOR signaling also reduces myeloid-derived suppressor cell production, also thought to play a role post-irradiation immune suppression against infection (66). Downregulation of P70S6K after MSC-EV treatment compared to untreated mice also suggests that mTOR activation has been reduced (67). We have shown that the mTOR/PPARγ axis is partly responsible for the acute and chronic (analogous to DEARE) immune dysfunction in burn injury (68–70), and experiments are underway to determine if MSC-EV can modulate this response in an mTOR-dependent fashion. It is therefore tempting to speculate that a key component of the reprogramming capacity of MSC-EVs is mTOR dependent. We found that MSC-EVs were also able to re-program immune response in a more severe polytrauma model (RCI), previously published findings demonstrate a greater amplitude of immune dysfunction compared to WBIR or burn alone (10), and were able to partially restore systemic cytokine patterns. We are currently testing the potential therapeutic use of MSC-EV in burn injury monotrauma models. While MSC-EV treatment has shown to be effective for mitigating the harmful effects of WBIR, we wanted to assess if MSC-EVs can simultaneously act as a radioprotectant for exposure to lethal doses of irradiation. We found that MSC-EVs administered prophylactically before exposure to 9 Gy of WBIR prolonged survival and mitigate weight loss (**Figure 5**). Due to the potential use of nuclear weapons in conflict and nuclear power plant accidents, there is a need to identify therapeutics that protect soldiers, emergency responders, and nuclear power plant workers to allow them to perform their duties in these hazardous environments. In addition, many cancer patients undergoing radiotherapy have to undergo less intense treatment plans, which are less effective for treating cancers, due to radiation toxicity. The use of a radioprotectant in these situations would be extremely beneficial as it would allow cancer patients to undergo more rigorous radiotherapy treatment regimens (31, 71). Future work will investigate the effects of combined pre-treatment and post-treatment of MSC-EVs, as well as their effects on cellular survival.

In November 2020, Klyachko et al. (72) reported that *clinicaltrials.gov* contained ~180 studies involving EVs as interventions or as a study object. Among these clinical trials, multiple timings and routes of administration were being utilized including oral, inhalation, nasal drop, i.v., and topical (72). As i.v. administration does not lend itself to being easily administered in the field, further work is required to assess the optimal dosage, efficacy of oral, intramuscular (i.m.) and intraperitoneal (i.p.) administration of MSC-EV in alleviating ARS and ultimately, DEARE. We present foundational data demonstrating the use of MSC-EV as a prophylactic therapy. Further experiments are in progress to examine the cellular and molecular mechanisms behind the increased survival after MSC-EV therapy before injury; regardless, these findings present an exciting avenue for the use of MSC-EVs in various applications, such as their use by military, firefighters and radiotherapy patients, and possibly in environmental situations to mitigate accrual of the damaging effects of low-level IR occupational exposure.

## Conclusions

Here, we demonstrate that proteomic and miRNA cargo of WBIR-induced EVs is altered depending on the dose and time after exposure. These EVs produce functional effects that are consistent with similar immune alterations observed in ARS. Lastly, we demonstrate that MSC-EVs can restore immune homeostasis following radiological injury and for the first time demonstrate that MSC-EVs can act as a radioprotectant.

## Data availability statement

The data presented in the study are deposited in the NIH GEO repository, accession numbers GSE234375, GSE233688 and GSE233692. The mass spectrometry proteomics data was deposited in the PRIDE database with the dataset identifier PXD041837.

## Ethics statement

The animal study was reviewed and approved by UNC Institutional Animal Care and Use Committee (IACUC).

## Author contributions

Conceptualization: RM, RS. Methodology: RM, LC, RS, SW, MW, LH, AM. Investigation: RM, LC, RS, LH, AM. Visualization: RM, LC, RS. Funding acquisitions: RM, LC, SW. Project administration: RM, LC, SW. Supervision: RM, LC. Writing-original draft: RM, LC, RS. Writing – review & editing: RM, LC, RS. All authors contributed to the article and approved the submitted version.

## Supplementary material

Supplementary Figure 1 Alterations in EV proteomic cargo following WBIR impact multiple physiological pathway categories. From the proteomic data, IPA was utilized to generate bubble charts displaying the general pathway categories that were most impacted. The size of the bubble denotes the number of proteins that overlap with that pathway and pink colors are associated with the activation of these pathways and blue is indicative of an inhibition of that pathway. **(A)** Bubble chart displaying the canonical pathways impacted based on the proteomic cargo of EVs released 3 days after 2 Gy of WBIR. **(B)** Bubble chart displaying the canonical pathways impacted based on the proteomic cargo of EVs released 7 days after 2 Gy of WBIR. **(C)** Bubble chart displaying the canonical pathways impacted based on the proteomic cargo of EVs released 3 days after 9 Gy of WBIR. Bubble chart displaying the canonical pathways impacted based on the proteomic cargo of EVs released 7 days after 9 Gy of WBIR. (E) Enzyme linked-immunosorbent assay (ELISA) for lymphocyte cytosolic protein-2 (LCP2) content in extracellular vesicles isolated 3 and 7 days after 9 Gy of WBIR.

Supplementary Figure 2 EVs released following WBIR induce immune gene expression changes in the absence of LPS. **(A)** Volcano plots displaying the immune gene changes in RAW macrophages exposed to EVs isolated 3 days after 9 Gy of WBIR compared to Sham EVs (n=3 for each experimental group). **(B)** Volcano plots displaying the immune gene changes in RAW macrophages exposed to EVs isolated 7 days after 9 Gy of WBIR compared to Sham EVs (n=3 for each experimental group). Canonical immune pathways identified to be most impacted by IPA with their associated -log10 (p-values).
