# Supplementary figures and images for: Involvement of extracellular vesicles in the progression, diagnosis, treatment, and prevention of whole-body ionizing radiation-induced immune dysfunction
Source: Front Immunol. 2023 Jun 15;14:1188830. doi: 10.3389/fimmu.2023.1188830 (PMC10316130; doi:10.3389/fimmu.2023.1188830)

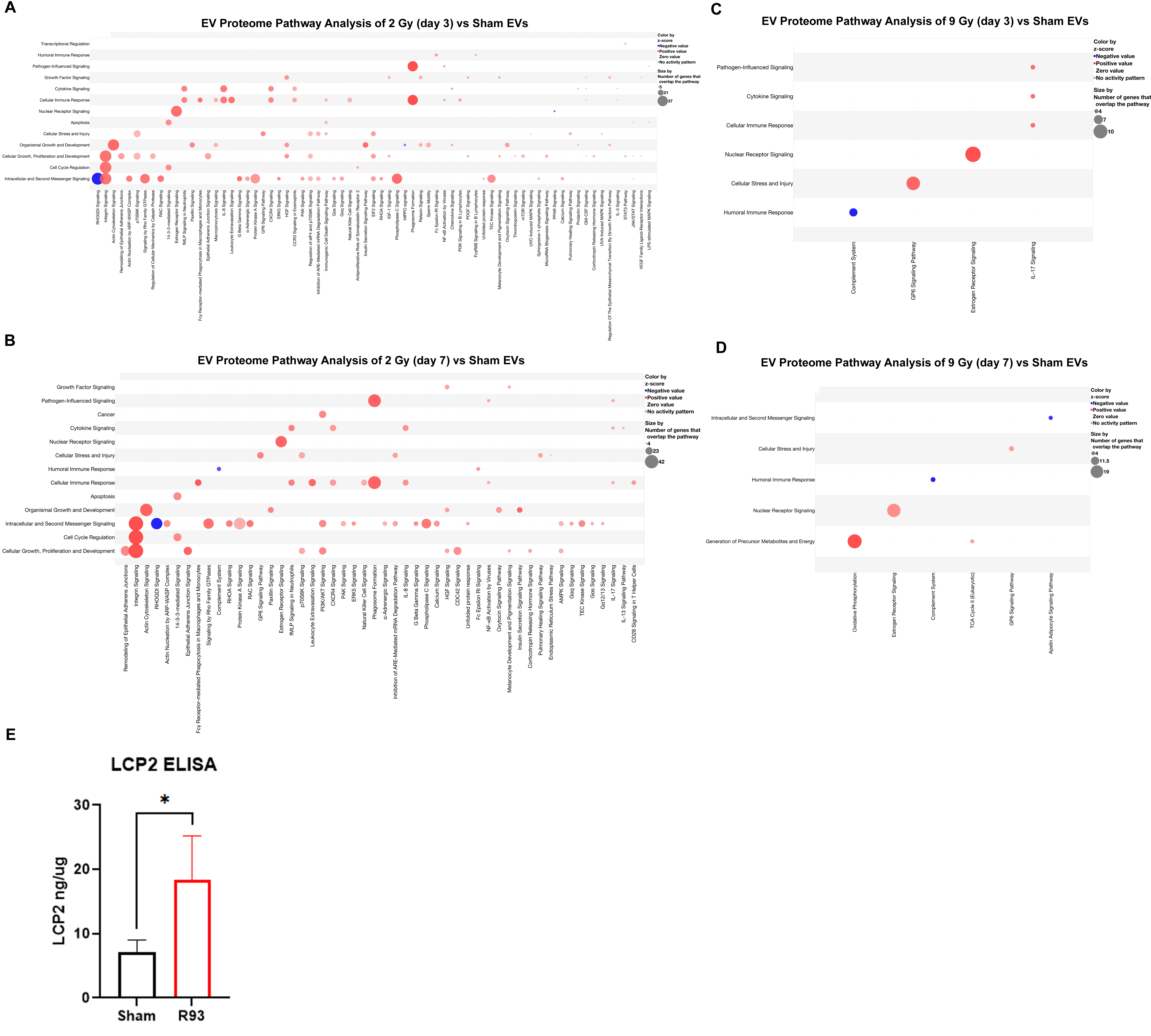

Supplement: Supplementary Figure 1 — Alterations in EV proteomic cargo following WBIR impact multiple physiological pathway categories. From the proteomic data, IPA was utilized to generate bubble charts displaying the general pathway categories that were most impacted. The size of the bubble denotes the number of proteins that overlap with that pathway and pink colors are associated with the activation of these pathways and blue is indicative of an inhibition of that pathway. (A) Bubble chart displaying the canonical pathways impacted based on the proteomic cargo of EVs released 3 days after 2 Gy of WBIR. (B) Bubble chart displaying the canonical pathways impacted based on the proteomic cargo of EVs released 7 days after 2 Gy of WBIR. (C) Bubble chart displaying the canonical pathways impacted based on the proteomic cargo of EVs released 3 days after 9 Gy of WBIR. Bubble chart displaying the canonical pathways impacted based on the proteomic cargo of EVs released 7 days after 9 Gy of WBIR. (E) Enzyme linked-immunosorbent assay (ELISA) for lymphocyte cytosolic protein-2 (LCP2) content in extracellular vesicles isolated 3 and 7 days after 9 Gy of WBIR. [file Image_1.jpeg]

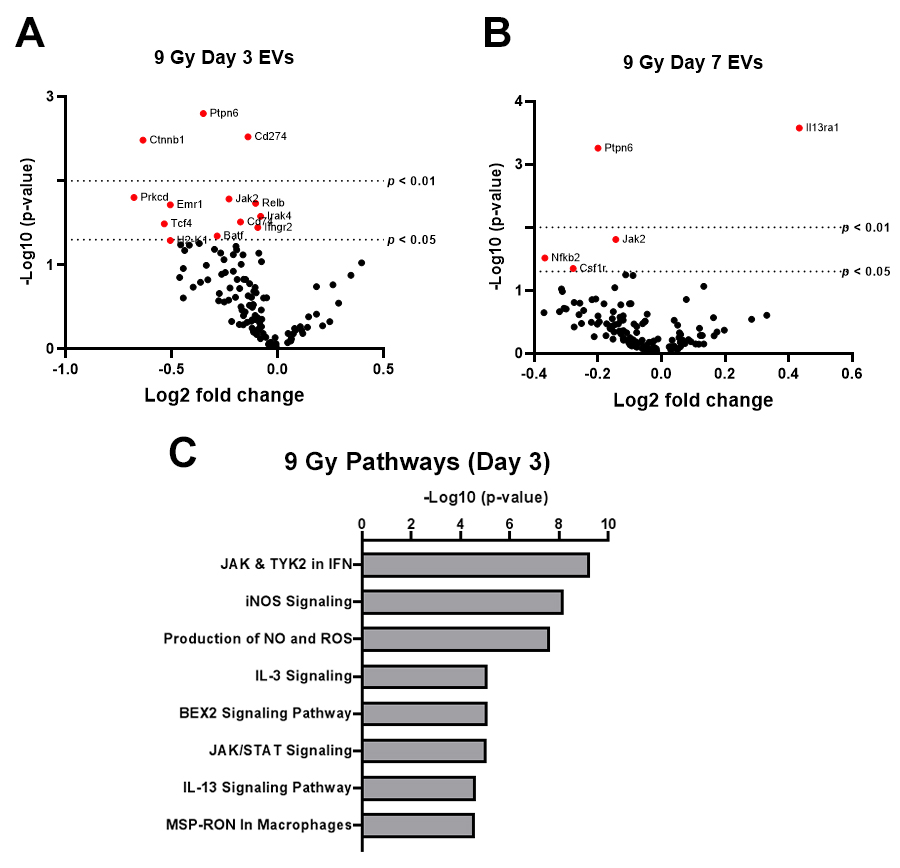

Supplement: Supplementary Figure 2 — EVs released following WBIR induce immune gene expression changes in the absence of LPS. (A) Volcano plots displaying the immune gene changes in RAW macrophages exposed to EVs isolated 3 days after 9 Gy of WBIR compared to Sham EVs (n=3 for each experimental group). (B) Volcano plots displaying the immune gene changes in RAW macrophages exposed to EVs isolated 7 days after 9 Gy of WBIR compared to Sham EVs (n=3 for each experimental group). Canonical immune pathways identified to be most impacted by IPA with their associated -log10 (p-values). [file Image_2.jpeg]
